# Análisis de narrativas del personal prestador de servicios anticonceptivos en México: entre la autonomía reproductiva y la coerción anticonceptiva

**DOI:** 10.1590/0102-311XES103324

**Published:** 2025-03-31

**Authors:** Ximena Quintero-Veloz, Janelli Vallin, Lauren Suchman, Kelsey Holt

**Affiliations:** 1 Universidad Nacional Autónoma de México, Ciudad de México, México.; 2 University of California San Francisco, San Francisco, U.S.A.

**Keywords:** Anticoncepción, Autonomía, Coerción, Paternalismo, Planificación Familiar, Contraception, Personal Autonomy, Coercion, Paternalism, Family Development Planning, Anticoncepção, Autonomia, Coerção, Paternalismo, Planejamento Familiar

## Abstract

O objetivo deste artigo foi identificar os relatos sobre autonomia reprodutiva e coerção contraceptiva de prestadores de serviços de planejamento familiar e contracepção, da equipe administrativa e dos criadores/operadores de políticas públicas em saúde reprodutiva a partir do referencial teórico da qualidade em assessoramento contraceptivo. Se trata de um estudo exploratório transversal, de natureza qualitativa. Realizamos 25 entrevistas semiestruturadas a 15 instituições de saúde pública, localizadas em áreas rurais, urbanas e de comunidades indígenas na Cidade do México e em San Luis Potosí, México. O método utilizado foi não probabilístico por conveniência e intencional para garantir a diversidade dos participantes. Realizamos uma análise de conteúdo dedutiva e indutiva por meio da qual identificamos um conjunto de relatos sobre autonomia reprodutiva, coerção contraceptiva e paternalismo. Os relatos sobre autonomia reprodutiva foram observados com maior frequência na Cidade do México, em mulheres, com graduação em medicina, em centros de saúde e em áreas urbanas. Os relatos com algum nível de coerção ou paternalismo foram citados com maior frequência em San Luis Potosí, por mulheres, com graduação em Medicina e Enfermagem, em centros de saúde e em áreas rurais. Os relatos paternalistas e coercitivos representam um problema que prejudica a qualidade das informações e serviços contraceptivos. Para mitigar esse problema e promover a autonomia reprodutiva, as entidades de saúde poderiam adaptar a estrutura da qualidade em assessoramento contraceptivo e incorporar a *Escala de Qualidade em Assessoramento Contraceptivo* ao sistema de saúde pública do México.

## Abstract (pt)

El objetivo de este artículo fue conocer las narrativas de autonomía reproductiva y coerción anticonceptiva de prestadores de servicios de planificación familiar y anticoncepción, personal administrativo y diseñador/operador de políticas públicas en salud reproductiva desde el marco teórico de la calidad en la consejería anticonceptiva. Seguimos un diseño de investigación cualitativo de corte transversal exploratorio. Realizamos 25 entrevistas semiestructuradas en 15 instancias públicas de salud situadas en contextos rurales, urbanos y pueblos originarios en Ciudad de México y San Luis Potosí, México. El método de muestreo fue no probabilístico por conveniencia e intencional para garantizar la diversidad de los participantes. Realizamos un análisis de contenido deductivo e inductivo por medio del cual identificamos un espectro de narrativas de autonomía reproductiva, coerción anticonceptiva y paternalismo. Las narrativas de autonomía reproductiva fueron emitidas con mayor frecuencia en la Ciudad de México, por mujeres, con nivel educativo de licenciatura en medicina; en centros de salud y en contextos urbanos Las narrativas con algún grado de coerción o paternalismo se pronunciaron con mayor frecuencia en San Luis Potosí; por mujeres, con nivel educativo de licenciatura en Medicina y Enfermería, en centros de salud y en contextos rurales. Las narrativas paternalistas y coercitivas representan un problema que socava la calidad de la información y los servicios anticonceptivos. Para mitigar este problema y fomentar la autonomía reproductiva, sugerimos a las autoridades sanitarias adaptar el marco teórico de la Calidad en Consejería Anticonceptiva así como incorporar la *Escala de Calidad en la Consejería Anticonceptiva* en el sistema público de salud en México.

## Abstract (en)

This study aimed to identify the narratives on reproductive autonomy and contraceptive coercion of family planning and contraceptive service providers, of administrative staff, and of the creators/operators of public policies in reproductive health based on the theoretical framework of quality of contraceptive counseling. This is a cross-sectional, exploratory and qualitative study. A total of 25 semi-structured interviews were conducted with 15 public health institutions located in rural, urban, and Indigenous communities in Mexico City and San Luis Potosí, México. Non-probabilistic convenience sampling was employed to ensure participants diversity. A deductive and inductive content analysis was conducted. The analysis enabled the identification of a set of narratives on reproductive autonomy, contraceptive coercion, and paternalism. Narratives on reproductive autonomy were observed more frequently in Mexico City, among women, with medical degrees, in health facilities, and in urban areas. Narratives with some degree of coercion or paternalism were cited most frequently in San Luis Potosí, by women, with degrees in Medicine and Nursing, in health facilities, and in rural areas. Paternalistic and coercive narratives represent a problem that impairs the quality of contraceptive information and services. To mitigate this problem and promote reproductive autonomy, health entities could adapt the quality of contraceptive counseling framework and incorporate the *Quality of Contraceptive Counselling* (QCC) *Scale* into Mexico’s public healthcare system.

## Introducción

El uso de métodos anticonceptivos es una práctica que puede fomentar la autonomía reproductiva. Sin embargo, las situaciones de abuso, maltrato o violencia que las mujeres viven dentro de las instituciones de salud en México están íntimamente relacionadas con los servicios ginecobstétricos y de salud reproductiva ^1^. A pesar de que las políticas y lineamientos vigentes promueven altos estándares de calidad en la atención de estos servicios para fomentar la autonomía reproductiva de las mujeres ^2^ ^,^ ^3^, existen narrativas en las instituciones de salud pública que obstaculizan los derechos reproductivos de las usuarias y, por lo tanto, disminuyen la calidad de los servicios.

Según la *Encuesta Nacional sobre la Dinámica de las Relaciones en los Hogares*, de 110.127 mujeres entre 15 y 49 años que tuvieron algún embarazo durante los cinco años anteriores, a 747 les colocaron un método anticonceptivo o las esterilizaron sin informarles; a 1.858 las presionaron para aceptar un dispositivo o la esterilización y a 245 las obligaron o amenazaron para que firmaran un papel sin información previa ^4^. Estos datos únicamente dan cuenta de la coerción que vivieron las mujeres para aceptar un método anticonceptivo o la esterilización en un contexto hospitalario post evento obstétrico, pero los resultados de la encuesta no permiten desagregar cada situación ni saber en qué otros contextos las mujeres enfrentan escenarios de coerción anticonceptiva.

Por otro lado, según datos de la Comisión Nacional de Arbitraje Médico, en el periodo de 2002 a 2017, la especialidad de ginecobstetricia ocupó el cuarto lugar con mayor número de quejas (3.090) ^5^. El motivo con mayor número de quejas fue la relación médico-paciente (1.130), entre las cuales se destaca la falta de información (393), información errónea o incompleta (473) y maltrato (264).

Si bien estos datos se publican para “*contribuir a la mejora de los procesos de calidad y seguridad del paciente al interior de los establecimientos e instituciones del Sistema Nacional de Salud*” ^5^ (p. 7), evidencian un problema que ha sido poco abordado en México: la evaluación de la calidad de los servicios anticonceptivos y de planificación familiar, específicamente de la provisión de orientación consejería y métodos anticonceptivos en distintos contextos médicos ^6^ ^,^ ^7^ ^,^ ^8^. Además, el corpus de estudios exploratorios de las narrativas de prestadores de servicios anticonceptivos ^1^ ^,^ ^8^ ^,^ ^9^ ^,^ ^10^ ^,^ ^11^ ^,^ ^12^, personal administrativo y autoridades sanitarias ^8^ aún es escaso, sin embargo, conocer su perspectiva es crucial para garantizar los más altos estándares de calidad de los servicios e identificar los aspectos que la debilitan.

En México se ha investigado la anticoncepción forzada como una forma de violencia obstétrica ^13^ ^,^ ^14^, no obstante, este concepto tiende a diluir formas más sutiles de coerción que las mujeres podrían enfrentar en otros servicios de salud pública ^1^ ^,^ ^10^ ^,^ ^12^. Asimismo, pocos estudios han visibilizado las narrativas del personal que fomentan la autonomía anticonceptiva de las usuarias ^8^.

Para contribuir a llenar estos vacíos, el objetivo de este estudio fue analizar las narrativas de autonomía reproductiva y coerción anticonceptiva de prestadores de servicios de planificación familiar y anticoncepción (PSPFA), miembros del personal administrativo (PA) y diseñador/operador de políticas públicas en salud reproductiva (DOPPSR) en dos estados mexicanos desde el marco teórico de la calidad en la consejería anticonceptiva. Dicha perspectiva teórica está construida para encuadrar la orientación consejería anticonceptiva de alta calidad centrada en la persona, basada en los derechos humanos y en la comunicación entre usuarias y PSPFA ^15^. Por ende, provee una guía para identificar los componentes de la orientación consejería que deben estar presentes para fomentar la autonomía y disminuir la coerción.

## Metodología

Este artículo sigue un diseño de investigación cualitativo de corte transversal y exploratorio. Se desprende de un proyecto de investigación más extenso que busca desarrollar, validar, y socializar la *Escala de Calidad en la Consejería Anticonceptiva* (*Quality of Contraceptive Counselling* -QCC- *Scale*) como una herramienta capaz de cuantificar la experiencia de las usuarias con la consejería en planificación familiar y anticoncepción en India, Etiopía y México ^7^. En México, realizamos 25 entrevistas de septiembre de 2020 a febrero de 2021 en colaboración con una asociación civil local.

Empleamos los métodos de muestreo no probabilístico por conveniencia e intencional. El muestreo por conveniencia se emplea para seleccionar a participantes que cumplan con un perfil establecido por el diseño de investigación; como suele componerse de poblaciones cautivas, se invita a quienes estén disponibles al momento del levantamiento de datos y su participación siempre es voluntaria ^16^ ^,^ ^17^. En esta investigación, construimos el perfil de participación de acuerdo con las funciones de tres actores clave en el campo de la planificación familiar y anticoncepción en el sistema de salud público mexicano: PSPFA, PA y DOPPSR. La muestra fue intencional porque buscamos que los participantes tuvieran la mayor diversidad posible en cuanto a sexo, edad, formación académica y funciones en distintas unidades sanitarias localizadas en diferentes contextos geográficos, económicos, políticos y socioculturales. Con ello, buscamos identificar cuál era el perfil del personal sanitario y en qué contextos tendrían mayor fomento en la autonomía o coerción en la prestación, administración y operación de políticas públicas de los servicios de planificación familiar y anticoncepción.

Para lograr la diversidad de la muestra, la seleccionamos en dos estados mexicanos con importantes contrastes, cuyas principales estadísticas pueden consultarse en la Tabla 1. Por un lado, la Ciudad de México es la capital federal, así como la ciudad más densamente poblada (6.163 habitantes/km^2^) ^18^ del país. Se localiza en la región centro-sur de México. La población capitalina tiene una edad mediana de 35 años, alta disponibilidad de servicios básicos y de tecnologías de la información y las comunicaciones (TICs), alta tasa de alfabetización, aunque con un porcentaje medio de afiliación a servicios de salud. Cuenta con una población católica a la baja y sin religión al alza ^18^. Tiene el índice de desarrollo humano -IDH- más alto del país (0,830) ^19^ y ha sido gobernada por partidos políticos progresistas. Si bien en la Ciudad de México hay un pequeño porcentaje de población hablante de lengua indígena (1,4%) y solo el 1% de la población vive en localidades rurales ^18^, cuenta con 139 pueblos originarios, es decir, aquellos de origen precolonial que conservan su identidad étnica e instituciones sociales, económicas, culturales y políticas ^20^. En contraste, San Luis Potosí es un estado ubicado en la región centro norte del país, con baja densidad poblacional (46 habitantes/km^2^) ^18^. La población potosina cuenta con edad mediana de 29 años, menor disponibilidad de servicios básicos y TICs, menor tasa de alfabetización, aunque con un porcentaje alto de afiliación a servicios de salud y de población católica y un bajo porcentaje sin adscripción religiosa ^18^. Su IDH es medio (0,726) ^19^ y ha sido gobernado por partidos políticos conservadores. El 8,6 % de la población es hablante de lengua indígena y el 33% vive en localidades rurales ^18^.

**Tabla 1:** Datos geográficos, sociodemográficos y políticos de Ciudad de México y San Luis Potosí, México.

Datos geográficos y sociodemográficos	Ciudad de México	San Luis Potosí
Población (n)	9.209.944	2.822.255
Superficie (km^2^)	1.494	61.138
Densidad poblacional (habitantes/km^2^)	6.163	46
Edad mediana (años)	35	29
Disponibilidad de servicios (%)		
Agua entubada	90,5	69,6
Drenaje	99,7	90,8
Servicio sanitario	99,7	98,0
Energía eléctrica	99,8	98,5
Disponibilidad de TICs (%)		
Computadora	59,9	34,0
Teléfono celular	92,2	84,5
Internet	75,7	44,5
Población hablante de lengua indígena (%)	1,4	8,6
Localidades (n)		
Urbanas	35	64
Rurales	599	6.490
Población que vive en localidades (%)		
Urbanas	99,0	67,0
Rurales	1,0	33,0
Tasa de alfabetización (%)		
15-24	99,2	98,9
25 y más	97,9	93,5
Afiliación a servicios de salud (%)	75,0	82,0
Religión (%)		
Católica	75,9	85,6
Protestante/Cristiano evangélico	7,3	8,1
Religiones diferentes	0,6	0,1
Sin religión	16,0	6,0
Partidos políticos en gobierno estatal	Partido de la Revolución Democrática (PRD): 1997-2018 Movimiento de Regeneración Nacional (MORENA): 2018-actualidad	Partido Revolucionario Institucional (PRI): 1997-2003, 2009-2021 Partido Acción Nacional (PAN): 2003-2009 Partido Verde Ecologista de México (PVEM): 2021-actualidad

TICs: tecnologías de la información y las comunicaciones.

Fuente: Instituto Nacional de Estadística y Geografía ^18^; Programa de las Naciones Unidas para el Desarrollo ^19^; Conferencia Nacional de Gobernadores ^36^.

Seleccionamos 15 unidades sanitarias: 2 centros de salud en localidades urbanas y 3 en pueblos originarios en Ciudad de México; 4 centros de salud y 2 hospitales en localidades urbanas y rurales en San Luis Potosí; una jurisdicción sanitaria y una oficina federal/estatal en cada estado (Tabla 2).

Entrevistamos a 25 participantes entre 19 y 66 años de edad, con antigüedad en su puesto de 3 meses a 11 años. Quince de ellos eran PSPFA, es decir, 5 enfermeras, 2 trabajadoras sociales y 2 promotores de salud, todos proveedores de información u orientación consejería en anticoncepción y 6 médicas que además realizaban la colocación, cambio o retiro de metodos anticonceptivos. El grupo de 8 administradores (PA) incluyó a un director de un centro de salud, 3 coordinadores médicos, 2 responsables jurisdiccionales de programas de salud sexual y reproductiva, un responsable jurisdiccional de farmacias y una asistente de control de insumos. Por último, entrevistamos a una persona DOPPSR en cada estado (Tabla 3).

**Tabla 2:** Características de las unidades de salud y contexto de los participantes. México.

Unidad de salud/Contexto	Ciudad de México	San Luis Potosí
Centro de salud		
Urbano	2	
Rural-urbano		4
Pueblo originario	3	
Hospitales		
Rural-urbano		2
Jurisdiciones sanitarias		
Urbano	1	1
Dirección federal/estatal		
Urbano	1	1

**Tabla 3:** Características del personal prestador de servicios de planificación familiar y anticoncepción entrevistado. México.

Características	Ciudad de México	San Luis Potosí	Total
Edad (19-66 años)	X̅ = 37,6	X̅ = 43,6	X̅ = 40
Sexo			
Mujer	12	6	18
Hombre	3	4	7
Nivel educativo			
Bachillerato/Técnico	3	1	4
Licenciatura	7	7	14
Posgrado	5	2	7
Formación			
Medicina	9	4	13
Enfermería	3	3	6
Trabajo social	1	1	2
Promoción de la salud		2	2
Ciencias Sociales	1	-	1
Bachillerato	1	-	1
Puesto			
Prestador de servicios de salud	8	7	15
Administrador/a	6	2	8
Operación de políticas públicas	1	1	2
Unidad de salud			
Centro de salud	11	5	16
Hospital comunitario	-	3	3
Jurisdicción sanitaria	3	1	4
Nivel federal/estatal	1	1	2
Contexto			
Urbano	8	2	10
Pueblo originario-urbano	7	-	7
Rural-urbano	-	8	8

Como herramienta de recolección de datos empleamos la entrevista semiestructurada, no obstante, las guías y el enfoque de la entrevista eran lo suficientemente flexibles para profundizar en temas que no estaban contemplados pero los participantes destacaban como importantes. Empleamos tres guías de entrevista, cada una dirigida a las tres poblaciones objetivo (Material Suplementario -Figuras S1, S2 y S3; https://cadernos.ensp.fiocruz.br/static//arquivo/supl-e00103324_6750.pdf). La guía dirigida a PSPFA exploró su experiencia respecto a la provisión de información, orientación consejería y servicios amigables para adolescentes; aplicación y cambio o retiro de un método anticonceptivo. La guía dirigida al PA estaba enfocada en la gestión y monitoreo de los servicios a nivel unidad de salud o jurisdiccional. Y con la guía dirigida a DOPPSR examinamos la experiencia de esta población, el diseño, implementación y supervisión de políticas públicas a niveles estatal y federal.

La coordinadora del proyecto en México, residente de la Ciudad de México, hablante nativa del idioma español, especializada en investigación cualitativa y, en ese momento, asistente de investigación recibió entrenamiento de la investigadora principal para adaptar e implementar las guías de entrevista al contexto mexicano.

Dado que realizamos el levantamiento de datos durante la tercera etapa de la pandemia por COVID-19, tomamos las medidas preventivas pertinentes en conjunto con las autoridades sanitarias para evitar el contagio epidémico. Por este motivo, implementamos las entrevistas en San Luis Potosí vía telefónica. En la Ciudad de México las realizamos en el consultorio u oficina de los PSPFA y PA; tanto entrevistada como entrevistados usaron cubrebocas y mantuvieron el distanciamiento físico en todo momento.

Antes de iniciar cada entrevista, presentamos a los participantes potenciales un consentimiento informado tanto para participar en el estudio como para grabar en formato audio. Quienes consintieron ambas cuestiones lo manifestaron de manera verbal al iniciar la entrevista y por escrito, ya fuera vía correo electrónico (San Luis Potosí) o con su firma autógrafa (Ciudad de México). Todas las entrevistas tuvieron una duración de 30 a 60 minutos.

Como muestra de agradecimiento, todos los participantes recibieron un incentivo con valor de USD 4,00. Además, entregamos al responsable de cada unidad de salud participante un paquete de materiales educativos sobre derechos sexuales y reproductivos. Si bien el Reglamento de la Ley General de Salud en Materia de Investigación para la Salud no contempla la entrega de incentivos a los participantes de una investigación ^21^, la Comisión Nacional de Bioética ^22^ aprueba la entrega de una retribución siempre y cuando se manifieste en el consentimiento informado, lo cual estaba presente por indicaciones de los comités de ética universitario y local.

Para garantizar la confidencialidad de los participantes, transcribimos todas las entrevistas, eliminamos los archivos de audio y almacenamos las transcripciones en una plataforma segura. Ninguna entrevista fue asociada con el nombre, localidad o centro de trabajo de los participantes.

El análisis de las transcripciones fue realizado por las dos primeras autoras con el programa Dedoose siguiendo el método de análisis de contenido con enfoque deductivo e inductivo. El análisis deductivo se fundamentó en el marco teórico de la calidad de la consejería anticonceptiva, el cual contempla tres fases de la orientación consejería: (1) evaluación de necesidades, (2) apoyo en la toma de decisiones y (3) elección del método y seguimiento. Las tres fases están fundamentadas en principios de derechos humanos relevantes para las interacciones entre usuaria y PSPFA (privacidad, confidencialidad, no discriminación), así como en conceptos de la teoría de la comunicación sanitaria fundamentales para la calidad interpersonal de la atención (respeto, empatía, confianza) ^15^. Realizamos una etapa piloto durante la cual codificamos ocho entrevistas. Con base en los resultados preliminares y de los conceptos del marco teórico, construimos un libro de códigos y subcódigos, mismo que refinamos durante reuniones semanales. Procedimos a codificar el resto de las entrevistas. Tanto del proceso de codificación como de la escritura y sistematización de memorandos, emergieron las subcategorías (Material Suplementario -Cuadro S1; https://cadernos.ensp.fiocruz.br/static//arquivo/supl-e00103324_6750.pdf).

El protocolo para implementar este estudio fue revisado y aprobado por la Junta de Revisión Institucional de la Universidad (estudio #18-26963) y por el Comité de Ética del Hospital Juárez de México (HJM 0768/20-1). El nivel de riesgo de esta investigación avalado por ambas instituciones revisoras fue mínimo ^23^.

## Resultados y discusión

Identificamos dos grandes categorías de análisis: narrativas de autonomía y narrativas con coerción. En el primer caso, las narrativas del personal entrevistado se enmarcaron dentro de los estándares de calidad del marco teórico de la Calidad en Consejería Anticonceptiva; en el segundo caso, identificamos formas de coerción que socavan la calidad de los servicios.

### Narrativas de autonomía

Definimos la autonomía reproductiva como un constructo multidimensional que consiste en “*tener el poder de decidir y controlar asuntos asociados con el uso de anticonceptivos, el embarazo y la maternidad*” ^24^ (p. 20).

Detectamos narrativas de autonomía pronunciadas por las 25 personas entrevistadas. Este tipo de narrativas son emitidas con mayor frecuencia en la Ciudad de México, por mujeres prestadoras de servicios de planificación familiar y anticoncepción -en particular por personal médico; con nivel educativo de licenciatura; en centros de salud y en contextos urbanos.

## Evaluación de necesidades

Las personas entrevistadas tienen claro que los elementos que facilitan y fortalecen la comunicación son la empatía y disposición para escuchar a las usuarias. Esto es tan importante como proporcionar información apropiada, y tener un lugar que garantice la privacidad.

Para los PSPFA es importante explorar las preferencias de las usuarias durante la consulta, incluyendo si desea tener más hijos o no y el tiempo durante el cual desea usar el método. Señalan que hay mujeres que viven situaciones complejas y sopesan factores como el trabajo o tiempo que tienen para acudir a los servicios anticonceptivos. Esto demuestra que los PSPFA reconocen que un mismo método no será funcional para todas las mujeres.

“*Brindar información necesaria para que el paciente tome la decisión del método que más le convenga de acuerdo con sus necesidades, estilo de vida, estado de salud, perspectivas e, inclusive, si quiere retirarse este método, pueda volver a la fertilidad*” (PA, médico cirujano, 58 años, jurisdición sanitaria, contexto urbano, Ciudad de México).

### Apoyo en la toma de decisiones

Tanto PA como DOPPSR afirman que la comunicación entre usuaria y personal de salud no consiste en obligar a una usuaria a seleccionar un método, sino a proporcionarle información clara y directa. De hecho, algunos PSPFA manifestaron que su rol consiste en informar y orientar para que sea cada usuaria quien decida el método más conveniente para sí misma. Cuando generan confianza y empatía con las usuarias hay mayor posibilidad de que la persona regrese a darle seguimiento a su método y recomiende el servicio.

### Elección del método anticonceptivo y seguimiento

Proporcionar información adecuada y respetar las preferencias de las usuarias fomenta que tomen sus propias decisiones y ejerzan su autonomía ^15^. Algunos PSPFA destacaron la importancia de respetar los deseos de las usuarias y que ellas sean quienes elijan el método anticonceptivo. Por ejemplo, si desean embarazarse a corto plazo, cambiar o retirarse un método o, incluso, no usar ninguno. Algunos PSPFA están conscientes de que no todas las usuarias quieren o necesitan un método y eso es aceptable.

Pocos PSPFA muestran flexibilidad y conocimiento de los derechos de las usuarias para cambiar o interrumpir el uso de un método. En contraste, tanto el PA como DPPSR tienen una percepción sólida de la autonomía basada en el ejercicio de los derechos humanos:

“*El prestador de servicios debe tener la habilidad suficiente para guiar y orientar para que, de acuerdo con las características y necesidades* [de la usuaria]*, dar la mejor información, y al final respetar absolutamente su decisión*” (DOPPSR, maestría en Ciencias Sociales, 58 años, oficina federal, contexto urbano, Ciudad de México).

### Placer

Tres PSPFA afirmaron que las usuarias tienen derecho a decidir y disfrutar de su sexualidad sin ser juzgadas. Para concretarlo, son indispensables la planificación familiar, la información y una orientación consejería que derive en la elección del mejor método anticonceptivo para cada una de ellas.

“*Es algo muy importante disfrutar la sexualidad, independientemente si quieres o no tener hijos*” (PSPFA, médica cirujana, maestría, 33 años, centro de salud, pueblo originario, Ciudad de México).

Una PSPFA incluso mencionó que quiere que las usuarias hagan preguntas sobre cómo protegerse y tener placer al mismo tiempo.

“*Justo tuve una consulta en la mañana de planificación familiar. Aclaramos varias dudas de los métodos anticonceptivos que ella recibía y al último entró tanto en confianza que me preguntó: ‘y para hacer sexo oral, ¿cómo me puedo proteger?’. Todo el personal de salud debemos entender que la sociedad ya incluye muchas prácticas sexuales diversas*” (PSPFA, médica cirujana, 28 años, centro de salud, contexto urbano, Ciudad de México).

Esta cuestión coincide con una propuesta de modelo integral sobre consejería de anticoncepción post evento obstétrico, particularmente dirigido a adolescentes, en el cual los autores recomiendan incorporar temas complementarios a la anticoncepción como “*el placer, la responsabilidad y la importancia de una vida sexual plena, confiable y sin riesgo de un embarazo no deseado o infecciones de transmisión sexual*” ^8^ (p. 388). Esto, sumado a las preferencias y expectativas sobre los métodos anticonceptivos, vida reproductiva y proyecto de vida de las usuarias, no solo fomentaría su autonomía reproductiva, sino también mejorará la calidad de la consejería anticonceptiva ^8^ ^,^ ^15^.

(i) Servicios amigables para adolescentes

Algunos PSPFA reconocen la autonomía de los adolescentes para ejercer su sexualidad de manera responsable y placentera. Para fomentarla, tratan de tener un consultorio agradable con murales en las paredes y mensajes de privacidad. Todos los entrevistados afirmaron que la orientación consejería debe ser accesible para todas las personas en edad reproductiva y que las creencias de los PSPFA no deben influir en la elección de las usuarias.

Sin embargo, algunos administradores reconocieron que no todos los médicos piensan así, por lo cual buscan distintas estrategias para garantizar el acceso de todas las personas a los servicios de anticoncepción y planificación familiar, por ejemplo, rescindir el contrato de los PSPFA que obstaculizan el acceso o buscar personal aliado que no tenga conflicto en atender a adolescentes.

## Narrativas con coerción

Definimos coerción anticonceptiva como el comportamiento del personal sanitario cuando ejerce -con mayor o menor grado- la fuerza, violencia, intimidación, manipulación, engaño o condicionamiento de servicios o derechos para interferir con el proceso de toma de decisiones autónoma de una mujer respecto a su salud reproductiva, específicamente, en la elección, uso, cambio o interrupción de un método anticonceptivo ^25^ ^,^ ^26^ ^,^ ^27^. Es posible que la coerción se implemente de manera individual, colectiva o institucional (como política interna dentro de una unidad de salud o como política pública que rige los servicios anticonceptivos a nivel estatal o nacional) ^1^ ^,^ ^14^ ^,^ ^27^. Por otro lado, definimos el paternalismo como la interferencia contra la voluntad de una persona (o sin su consentimiento) para tomar decisiones o actuar de una manera específica “por su propio bien” o para que “no se haga daño a sí misma ni a outros” ^28^ ^,^ ^29^. Entendemos las narrativas paternalistas como las formas más sutiles dentro del espectro de la coerción.

En cada una de las etapas planteadas por el marco teórico de la Calidad en Consejería Anticonceptiva existen formas en las cuales los PSPFA pueden tener actitudes o narrativas coercitivas o paternalistas, por ejemplo, durante la evaluación de necesidades podrían no identificar las necesidades de cada usuaria; durante el proceso de toma de decisión podrían proporcionar información sesgada; durante la elección del método y seguimiento podrían ignorar la elección de la usuaria o inducirla a elegir un método cuando no desea usar uno o bien, forzarla a usar uno que no eligió.

Identificamos narrativas con algún grado de coerción o paternalismo emitidas por 23 de las 25 personas entrevistadas. Se pronunciaron con mayor frecuencia en San Luis Potosí; por mujeres; personal sanitario -en concreto, personal médico y de enfermería- con nivel educativo de licenciatura; em centro de salud y en un contexto rural.

### Definición y objetivo de la orientación consejería

En los servicios de salud analizados no existe un consenso oficial en torno a cómo se define una orientación consejería, qué elementos debe incorporar, quién puede proporcionarla o cuál es su objetivo en el ámbito de la anticoncepción. Esto da lugar a una multiplicidad de interpretaciones que dependen de la profesión y capacitación que el prestador de salud ha recibido sobre los servicios de planificación familiar, anticoncepción y derechos sexuales y reproductivos, en particular, de adolescentes.

Hubo quienes afirmaron que su papel como PSPFA en este ámbito es convencer a las usuarias de aceptar un metodo anticoncepcional. Esta interpretación resulta problemática porque fomenta prácticas paternalistas o coercitivas. Una de ellas, también identificada en otro estudio en un contexto hospitalario postparto en México ^1^, consiste en que distintos miembros del equipo sanitario presionan a la usuaria hasta que acepte un metodo anticonceptivo:

“*Primero, el área de consulta externa, después, las de enfermería, después yo también les tengo que dar un poquito más de orientación. Como a veces tenemos pacientes que no quieren, nos la vuelven a canalizar a trabajo social para volverlas a orientar. Y a veces sí nos funciona que sí las podemos convencer*” (PSPFA, trabajadora social, 34 años, hospital, contexto rural, San Luis Potosí).

Ofertar un metodo anticonceptivo durante las consultas médicas es una buena estrategia para difundir la información de este servicio entre las usuarias, pero puede volverse coercitiva o paternalista si es malinterpretada como que todas las mujeres en edad reproductiva deben usar un metodo anticonceptivo, de preferencia, de larga duración.

### Preferencias personales de prestadores de servicios anticonceptivos

En ocasiones, las preferencias personales de los PSPFA influyen en la información o metodos anticonceptivos que le proporcionan a la usuaria con base en prejuicios como la edad:

“[Mis compañeras] *les ponen, casi a todas, inyectable bimensual, y a mí no me gusta ese método. Entonces, me esperan para dar la consejería. Porque para mí es el implante si son adolescentes o DIUs hormonales si son más grandes*” (PSPFA, enfermera, maestría, 50 años, hospital, contexto rural, San Luis Potosí).

Algunos PSPFA consideran que los métodos reversibles de larga duración son más efectivos para planificar, por lo que en ocasiones los recomiendan más que a otros métodos anticonceptivos. Al respecto, encontramos el testimonio de una médica que desaconsejó el uso del parche a una usuaria argumentando que era un método poco seguro, a pesar de que la usuaria lo solicitó y no tenía ninguna contraindicación clínica para usarlo (médica cirujana, 33 años, centro de salud, pueblo originario, Ciudad de México). Con frecuencia, los PSPFA proporcionan información sesgada o fomentan el uso de unos métodos, pero omiten otros, aunque el PA y las DOPPSR se aseguran de que haya una alta disponibilidad y una extensa gama de métodos anticonceptivos disponibles en todas las unidades sanitarias. Este es un ejemplo de que un método anticoncetivo puede estar disponible en una unidad de salud, pero puede no ser accesible para una usuaria porque el personal presenta barreras basadas en sus propias preferencias o valoraciones para acceder a él, con lo cual, se incumplen las recomendaciones de la Organización Mundial de la Salud (OMS) para garantizar los derechos humanos de las usuarias de servicios anticonceptivos ^30^.

En este punto, nuestros hallazgos en torno a la coerción anticonceptiva, obtenidos desde el punto de vista del personal de salud, coinciden con otros estudios cualitativos centrados en las usuarias. Por ejemplo, un estudio realizado en Etiopía ^31^, reveló que las usuarias sintieron que los proveedores ignoraron sus preferencias en favor de los métodos anticonceptivos reversibles de larga duración. Otro estudio realizado con mujeres jóvenes de Guanajuato, México, demostró que a las usuarias se les negó el método que ellas preferían sin explicación alguna ^32^.

### Prejuicios de prestadores de servicios anticonceptivos en torno a las usuarias

Detectamos distintas condiciones con base en las cuales el personal ejerce algún grado de coerción para hacer que una usuaria acepte un metodo anticonceptivo: edad, número de hijos, embarazo y situación económica:

“*Depende mucho la edad de la paciente. Si es una adolescente que está estudiando o que no estudia pero que está en edad para que estudie, siempre la voy a orientar para que tenga una preparación académica más amplia, para que tenga un futuro, ya no para sus hijos, sino para ella misma primero. Si es una mujer añosa, una mujer de 35 en adelante y ya tiene dos, le voy a decir: ‘si usted, señora, ya tiene tal edad, debe de pensar en no tener un niño con síndrome de Down, debe de pensar en que no arriesgue su vida’.* (...) *Creo que he estado impactando en mi población porque cuando yo llegué a este módulo eran 600 mujeres -hace 2 o 3 años- ingresadas en el programa de planificación familiar. Ahora tengo 1.500 mujeres activas*” (PSPFA, enfermera, maestría, 50 años, hospital, contexto rural, San Luis Potosí).

En ocasiones, los PSPFA reciben mensajes contradictorios sobre cómo promover servicios de calidad de las políticas y metas numéricas. Mencionaron la importancia de asesorar a las mujeres de acuerdo con sus necesidades y respetar sus elecciones, pero afirmaron que una orientación consejería exitosa resultaría en que un mayor número de usuarias acepten el uso de un anticonceptivo o desistan de cambiarlo o retirarlo, lo cual ya ha sido documentado en otros estudios ^11^.

Sobre la misma línea, dos PSPFA entrevistadas manifestaron tratar de convencer a mujeres entre 15 y 25 años para que no traigan hijos a sufrir. Esta es una noción extendida en México basada en prejuicios en torno a la edad, clase social, situación económica o el contexto de la usuaria. Con estas narrativas, algunos PSPFA establecieron parámetros sobre los casos en los que las mujeres deberían abstenerse de tener hijos, no obstante, no mencionaron una situación o edad ideal para hacerlo. De todos los escenarios que plantearon, en su opinión, ninguna mujer estaba en condición de tener descendencia, ya fuera porque eran demasiado jóvenes, demasiado añosas, tenían ya demasiados hijos, no contaban con una formación académica aceptable o con un trabajo formal con la remuneración o los recursos materiales suficientes para ofrecer a su descendencia una vida digna. La perspectiva de estas PSPFA no solo resulta coercitiva y paternalista, también concuerda con el concepto de reproducción estratificada, con el cual Colen evidenció que la valoración social de la maternidad está sustentada en “*tareas reproductivas fisiológicas y sociales realizadas de manera diferencial según las desigualdades sustentadas en jerarquías de clase, raza, etnicidad, género, lugar en una economía global y estatus migratorio, estructuradas por fuerzas sociales, económicas y políticas*” ^33^ (p. 393). Desigualdades que, en este caso, son reproducidas en los servicios anticonceptivos del sistema de salud mexicano.

Por otro lado, algunas de las personas entrevistadas mencionaron la importancia de conocer los antecedentes ginecobstétricos, aunque en ocasiones relacionados con el comportamiento sexual de la usuaria, como el número de parejas sexuales que tiene o ha tenido. Si bien el lineamiento técnico para la prescripción y uso de métodos anticonceptivos en México ^2^ sugieren la recopilación de esta información, no es necesaria ni constituye un criterio de elegibilidad para ningún método de acuerdo con la OMS ^30^ ^,^ ^34^ ^,^ ^35^.

### Negativa de cambio o suspensión de un método anticonceptivo

Encontramos una postura extendida de desconfiar de las usuarias cuando reportan que están experimentando efectos secundarios. En ocasiones, las acusan de mentir (PSPFA, médica cirujana, 28 años, centro de salud, contexto urbano, Ciudad de México) o de poner pretextos para lograr que les retiren el método (PSPFA, promotor de salud, 53 años, centro de salud, contexto rural, San Luis Potosí).

Las prestadoras de salud suelen investigar sus síntomas desde una perspectiva coercitiva por medio de una exploración de cavidades o de estudios de laboratorio, como perfil hormonal y papanicolaou, pero si no encuentran un motivo real para retirarlo o cambiarlo, no lo hacen. Es destacable que la mayor parte del personal médico no solicita estudios de laboratorio ni realizan la exploración física para prescribir un método anticonceptivo por primera vez; solo realizan estos procedimientos para verificar que las usuarias estén diciendo la verdad respecto a los efectos adversos que experimentan, en particular si son adolescentes o mujeres en puerperio. Esta situación es relevante porque en ninguna de las guías de prestación de servicios anticonceptivos o planificación familiar se requiere de esos procedimientos para prescribir un metodo anticonceptivo ni para cambiarlo o suspenderlo.

Algunos PSPFA expresaron preocupación porque sus pares omiten informar a las usuarias sobre los efectos secundarios porque creen que podrían predisponer a las usuarias a sentir efectos secundarios que no tendrán. Una prestadora afirmó que, en su práctica cotidiana, es frecuente que atienda a usuarias a quienes se les colocó un método anticonceptivo de larga duración sin una orientación previa y sin su consentimiento en el hospital en donde atendieron su parto. En estos casos, esta enfermera accede a proporcionar la orientación consejería y respetar la decisión de la usuaria respecto al método que le fue colocado sin recibir información previa:

“*Bueno, esto nos pasa con frecuencia. Las pacientes vienen de un embarazo, en el hospital en donde se atendieron, ellas refieren que no se les da una orientación, simplemente se los colocan*” (PSPFA, técnica en Enfermería, 34 años, centro de salud, contexto urbano, Ciudad de México).

No obstante, no todo el personal de salud respeta las decisiones de las usuarias. Para algunos de ellos, esta situación implica una labor de convencimiento para que la usuaria no se retire o cambie el metodo anticonceptivo que le fue colocado sin su consentimiento. Parece que en los hospitales continúa la práctica médica autoritaria de la anticoncepción forzada para convencer a las mujeres que acaban de tener un parto para que acepten un metodo anticonceptivo de larga duración, lo cual coincide con los hallazgos de otros estudios, particularmente en un contexto hospitalario post evento obstétrico ^10^ ^,^ ^12^.

En estos casos, las usuarias podrían enfrentar dos escenarios de coerción: primero, durante el puerperio, cuando los PSPFA las presionan para aceptar un metodo anticonceptivo antes de darlas de alta y después, cuando desean retirárselo, para lo cual consultan a un PSPFA en una unidad de salud, pero se encuentran con una política extraoficial que las obliga a usarlo por un periodo mínimo de 6 meses a 3 años.

## Conclusiones

Las narrativas sostenidas por los PSPFA, PA y DOPPSR entrevistados se posicionan dentro de un amplio espectro que oscila entre la autonomía reproductiva y la coerción anticonceptiva.

Es destacable que todas las personas entrevistadas emitieron narrativas de autonomía en algún momento de la entrevista y tratan de fomentarlas en su práctica profesional cotidiana. Se resalta que los PSPFA fue el grupo que emitió más narrativas de autonomía en Ciudad de México y, al mismo tiempo, más narrativas coercitivas o paternalistas, particularmente médicas y enfermeras en los contextos rurales de San Luis Potosí. Si bien consideramos que las narrativas coercitivas son fomentadas por algunos prejuicios sobre las usuarias y poca capacitación específica en la prestación de orientación consejería de alta calidad centrada en la persona y desde una perspectiva de derechos humanos, también lo son por las metas numéricas que el personal de salud debe alcanzar respecto a número de usuarias o métodos anticonceptivos entregados.

Algunas acciones que podrían coadyuvar en la disminución de la coerción anticonceptiva y la promoción de la autonomía reproductiva de las mujeres mexicanas son definir del concepto y objetivo de la orientación consejería anticonceptiva, publicar manuales específicos para proporcionarla, y capacitar y sensibilizar al personal de salud. Es crucial desplazar el foco en metas numéricas hacia la incorporación de instrumentos de medición de la coerción y la calidad de la consejería anticonceptiva. Como alternativa, sugerimos retomar, traducir y adaptar el marco teórico de la Calidad en Consejería Anticonceptiva ^15^, así como implementar la *Escala de Calidad en la Consejería Anticonceptiva* en el sistema público de salud en México ^7^.
