# Supplementary material for: Analysis of the reports of contraceptive service providers in Mexico: between reproductive autonomy and contraceptive coercion
Source: Cad Saude Publica. 2025 Mar 31;41(2):e00103324. [Article in Spanish] doi: 10.1590/0102-311XES103324 (PMC11960756; doi:10.1590/0102-311XES103324)
Supplement: Supplementary file 1 [file 1678-4464-csp-41-02-ES103324-s.pdf]

Guía de entrevista para prestadores de servicios de planificación familiar y anticoncepción

Antecedentes:

1. Edad
2. Puesto que ocupa en este centro de salud
3. Tiempo en el puesto
4. Formación
5. ¿Qué tipo de capacitación en consejería anticonceptiva ha recibido?

Prestadores:

1. ¿En qué medida la consejería anticonceptiva es parte de su función como prestadora de servicios de salud?
   a. ¿Cuánto tiempo lleva proporcionando consejería anticonceptiva? (En este centro de salud o en otra unidad)
   b. ¿Con qué frecuencia habla de anticonceptivos con las usuarias?
   c. ¿Aproximadamente cuántas consultas sobre anticonceptivos atiende por día/semana?
   d. ¿Hay un tipo de usuarias que asista con mayor frecuencia para solicitar un método anticonceptivo?

2. Desde su perspectiva, ¿cuál es el objetivo de proporcionar consejería anticonceptiva a las usuarias?

3. ¿Cuál es su papel en la decisión que las usuarias toman?

Enfoque de consejería:

Ahora me gustaría hablar sobre el enfoque que utiliza cuando habla con una usuaria sobre anticoncepción. Sabemos que hay una gran cantidad de escenarios que enfrenta durante una orientación consejería de anticoncepción, voy a plantear algunos donde mujeres con diferentes necesidades recurren a usted para solicitar un anticonceptivo, por favor describa detalladamente cada proceso, o si lo prefiere, podemos simular que yo soy la usuaria en cada escenario y que usted me ayudaría a seleccionar un método:

1) Una mujer que recurre a usted para usar métodos anticonceptivos por **primera vez**.
   a. ¿Cómo la ayuda a seleccionar un método?

2) Una mujer que **ha usado anticonceptivos previamente**, pero **quiere cambiar de método** y ahora recurre a usted para obtener información.
   a. ¿Cómo la ayuda a seleccionar un método?
   b. ¿Es diferente a la que le proporciona a una mujer que usará métodos anticonceptivos por primera vez?
3) Una mujer que **solicita el retiro de un método de larga duración**.
   a. ¿Qué preguntas hace a la usuaria sobre sus razones para pedir que retiren su método de larga duración?
   b. ¿Qué hace si una mujer viene **poco tiempo después** de tener su implante o su DIU insertado para pedir que lo remuevan? (Por ejemplo: Una mujer que recibió un implante con duración de cinco años, viene 6 meses después para pedir que se lo

retiren)

4) Una mujer acude para dar **seguimiento a los efectos secundarios** con su método anticonceptivo
    a. ¿Qué información u orientación le da?

4. ¿Cómo puede saber si un método anticonceptivo es apropiado o no para una mujer específica?

5. Como prestadora de servicios de anticoncepción y planificación familiar, ¿cuáles son los factores que podrían afectar la forma en la que usted proporciona la orientación consejería?
    a. Sondear: Factores de nivel clínico tales como infraestructura/gestión/administración, política
    b. Sondear: Factores individuales tales como carga de trabajo, número de pacientes, agenda, acceso a capacitación, etc...

6. ¿Tiene el centro de salud un manual o guía escrita sobre la prestación de la consejería anticonceptiva?
    a. Sondear: ¿Cuáles son los contenidos clave?

General:

Evaluación y gestión de la calidad

Ahora me gustaría hacerle algunas preguntas sobre qué procesos de evaluación o gestión de la calidad deben existir para mejorar los servicios de anticoncepción y planificación familiar.

7. En su opinión, ¿considera que en este centro de salud se brinda una consejería anticonceptiva de alta calidad?
    a. ¿Por qué?
    b. ¿Cuáles son los aspectos que hacen que la consejería sea de alta calidad?

8. ¿Se ha llevado a cabo alguna actividad de gestión de la calidad para mejorar la orientación consejería y los servicios de anticoncepción y planificación familiar en este centro de salud?
    [No- pasar a la siguiente pregunta]
    [Sí]- dígame en detalle qué se realizó [y sondear]:
    a. ¿Quién llevó a cabo la gestión de calidad y quién colaboró?
    b. ¿Cuándo se hizo?
    c. ¿Qué personal del centro de salud se incluyó en las actividades de mejora de la calidad?
    d. ¿Cuáles fueron los puntos fuertes de este proceso?
    e. ¿Cuáles fueron las debilidades?
    f. ¿Cuáles fueron los desafíos en la implementación de actividades de mejora de la calidad?

9. Si se hiciera alguna actividad de gestión de la calidad, ¿qué aspectos de la consejería anticonceptiva podrían mejorarse en este Centro de Salud?

10. ¿Alguna vez ha recibido retroalimentación de las usuarias sobre la consejería anticonceptiva que usted proporcionó en este centro de salud?
    [No- pasar a la siguiente pregunta]
    [Sí- Sondear]:

a. Cuénteme cómo fue esa experiencia.
b. ¿Quién le proporcionó los comentarios?
c. ¿Cómo se los hizo llegar?
d. ¿Qué hizo en respuesta a los comentarios?
e. ¿Cómo fue esta experiencia para usted?

11. ¿Considera importante recibir retroalimentación de las usuarias sobre la forma en la que proporciona la consejería anticonceptiva?

12. ¿Cómo preferiría recibir estos comentarios? [De manera verbal, escrita, otra]
    ¿Por qué?

13. Planteamos estas preguntas porque las encuestas de experiencia de las usuarias son una forma de conocer su percepción de los servicios de anticoncepción y planificación familiar. ¿Cómo le parece la idea de recopilar comentarios de las usuarias a través de encuestas de experiencia para mejorar la calidad?

14. ¿Considera que este Centro de Salud cuenta con las condiciones idóneas para implementar una encuesta de experiencia para mejorar la calidad de la consejería anticonceptiva? ¿Qué tan factible es implementar una encuesta de experiencia dirigida a las usuarias?
    a. ¿Qué factores tendrían que estar presentes para que fuera posible?

15. ¿Cómo podrían utilizarse los resultados de esta encuesta dirigida a las usuarias para mejorar la calidad de los servicios de anticoncepción en este Centro de Salud?

16. Si se pudiera retroalimentar a las prestadoras de salud con estos resultados, ¿preferiría recibirla por medio de comentarios individualizados (a nivel de prestadora) o comentarios generales dirigidos al Centro de Salud?
    a. ¿Cómo se sentiría al saber que sus compañeras o supervisoras tendrían acceso a los resultados de su desempeño?

17. Si las usuarias reportaran experiencias negativas respecto a la atención anticonceptiva, ¿cómo podrían abordarse esos comentarios en su Centro de Salud?

18. ¿Alguna vez ha recibido comentarios del director del Centro de Salud, de la Jurisdicción Sanitaria o de la Secretaría de Salud sobre su práctica de consejería anticonceptiva? [Los comentarios podrían ser uno a uno a nivel personal o en grupo con otras prestadoras de planificación familiar.]
    [No- pasar a la siguiente pregunta]
    [Sí- Cuénteme cómo fue esta experiencia]. Sondear:
    a. ¿Quién le proporcionó los comentarios y cómo se entregó?
    b. ¿Qué le gustó y qué no le gustó de este proceso?
    c. ¿Respondió a los comentarios? ¿Cómo lo hizo?
    d. ¿Qué recomendaría al recibir comentarios en el futuro?

19. Si recibiera estos comentarios, ¿qué necesitaría para mejorar la atención anticonceptiva que proporciona en este centro de salud?
    a. **Sondear**: ¿En la gestión, la política, puntos públicos de entrega de servicios de salud?
    b. ¿Qué desafíos esperaría?
        i. ¿Por qué?

c. ¿A qué barreras se enfrenta para realizar este tipo de mejorías?
d. ¿Qué apoyo sería útil para realizar mejorías en este tipo de atención?

20. ¿Cómo es la planeación y evaluación del servicio anticonceptivo en este centro de salud? Cuénteme lo que conoce de estos procesos.
    a. ¿Quiénes lo realizan?
    b. ¿Planea usted proporcionar un número específico de anticonceptivos dentro de un mes o un año?
        i. **(Pregunte si hay un plan por tipo de usuaria, tipo de método anticonceptivo, etc.)**
        ii. ¿Cómo se establecen los números?
    c. Si tiene que seguir un plan o metas específicas, ¿cómo afecta esto en su prestación de servicios?

Figura S1 Guía de entrevista para prestadores de servicios de planificación familiar y anticoncepción (versión: 3 de noviembre de 2020).

Guía de entrevista para personal administrativo

Antecedentes:

1. Edad
2. Puesto que ocupa en este centro de salud
3. Tiempo en el puesto
4. Formación
5. ¿Qué tipo de capacitación en consejería anticonceptiva ha recibido? [indagar formación universitaria].

Preguntas generales

1. Desde su puesto, ¿de qué forma está involucrado en la prestación de servicios de anticoncepción y planificación familiar?

2. ¿Esta Unidad de Salud o los Centros de Salud bajo su jurisdicción cuentan con un manual o guía operativa escrito sobre consejería anticonceptiva?
   a. Sondear: ¿Cuáles son los contenidos clave?

Evaluación y gestión de la calidad

3. En su opinión, ¿cuál es el objetivo de la consejería anticonceptiva?

4. ¿Qué acciones realiza desde su puesto para asegurarse de que las usuarias reciban atención anticonceptiva de alta calidad?

5. ¿Cuál es su opinión sobre la calidad de la consejería anticonceptiva que se ofrece en esta **Unidad de Salud/en los Centros de Salud bajo su Jurisdicción**?
   a. Sondear: ¿Cuáles son algunos de los aspectos de la consejería anticonceptiva que usted considera que son de alta calidad?
   b. Sondear: ¿Qué aspectos de la consejería anticonceptiva podrían mejorar?
      i. ¿Por qué?

6. ¿Cómo se monitorea o evalúa el rendimiento de los prestadores de salud al proporcionar consejería anticonceptiva?

7. ¿Se ha llevado a cabo alguna actividad de gestión de la calidad implementada para mejorar la consejería anticonceptiva en esta unidad de salud?
   [No- pasar a la siguiente pregunta]
   [Sí]- dígame en detalle qué se realizó [y sondear]:
   a. Sondear: ¿quién llevó a cabo el trabajo de gestión de la calidad y quién colaboró?
   b. Sondear: ¿Cuándo se hizo?
   c. Sondear: ¿Qué personal del centro de salud se incluyó en las actividades de mejora de la calidad?
   d. Sondear: ¿Cuáles fueron los puntos fuertes de este proceso?
   e. Sondear: ¿Cuáles fueron las debilidades?
   f. Sondear: ¿Cuáles fueron los desafíos en la implementación de actividades de

mejora de la calidad?

8. ¿Qué necesita para realizar este tipo de mejoras de calidad a nivel administrativo o de dirección?
    a. ¿A qué barreras se enfrenta para realizar este tipo de mejoras?
    b. ¿Qué tipo de apoyo necesitaría para realizar este tipo de mejoras?

9. ¿Alguna vez ha recibido retroalimentación de las usuarias sobre la consejería anticonceptiva que se proporciona en este Centro de Salud/Jurisdicción?

    [No- pasar a la siguiente pregunta]

    [Sí- Sondear]:

    f. Cuénteme cómo fue esa experiencia.
    g. ¿Quién le proporcionó los comentarios?
    h. ¿Cómo se los hizo llegar?
    i. ¿Qué hizo en respuesta a los comentarios?
    j. ¿Cómo fue esta experiencia para usted?

10. ¿Considera importante recibir retroalimentación de las usuarias sobre la forma en la que se proporciona la consejería anticonceptiva en el sistema público de salud?

11. ¿Cómo preferiría recibir esta retroalimentación (de las usuarias sobre la consejería anticonceptiva)? [De manera verbal, escrita, otra]
    a. ¿Por qué?

12. Planteamos estas preguntas porque las encuestas de experiencia de las usuarias son una forma de conocer su percepción de los servicios de consejería anticonceptiva. ¿Cómo le parece la idea de recopilar comentarios de las usuarias a través de encuestas de experiencia para mejorar la calidad?

13. ¿Considera que este Centro de Salud cuenta con las condiciones idóneas para implementar una encuesta de experiencia para mejorar la calidad de la consejería anticonceptiva? ¿Qué tan factible es implementar una encuesta de experiencia dirigida a las usuarias?
    a. ¿Qué factores tendrían que estar presentes para que fuera posible?

14. ¿Cómo podrían utilizarse los resultados de la encuesta dirigida a las usuarias para mejorar la calidad de los servicios de anticoncepción en este Centro de Salud/Jurisdicción?

15. Si se pudiera retroalimentar a los prestadores de servicios de salud con estos resultados, ¿preferiría entregarla por medio de comentarios individualizados (a nivel de prestador) o comentarios generales dirigidos al Centro de Salud, por Jurisdicción o por región?

16. Si las usuarias reportaran experiencias negativas respecto a la atención anticonceptiva, ¿cómo podrían abordarse esos comentarios en su Centro de Salud/Jurisdicción?

17. ¿Cuál sería la mejor manera de entregar comentarios a las Unidades de Atención Médica sobre su desempeño de consejería anticonceptiva [de forma oral, escrita; en grupos, de forma individual; recomendaciones por unidad de salud, etc.]?
    a. ¿Cómo podría este proceso ser más fácil/mejor?

18. ¿Cómo es la planeación y evaluación del servicio anticonceptivo en este Centro de Salud/Jurisdicción?
    a. ¿Cómo se hacen los planes para el suministro de anticonceptivos?
    b. ¿Planea proporcionar un número específico de anticonceptivos dentro de un mes o un año? Cuénteme más al respecto
        i. ¿Cómo se establecen estos números?
        ii. ¿Qué hace para cumplir con su plan de provisión de anticonceptivos?

19. ¿Cómo se miden las metas de los servicios anticonceptivos y de planificación familiar (por ejemplo: en un periodo de tiempo en un mes, trimestre o año)?
    a. ¿El establecimiento de estas metas afecta de alguna manera a los prestadores? ¿Cómo?
    b. ¿Cómo se utiliza el desempeño de los prestadores en su administración [en el Centro de Salud, Jurisdicción Sanitaria o Secretaría de Salud]?

20. ¿La Secretaría de Salud o la Jurisdicción Sanitaria han hecho alguna vez una supervisión sobre la prestación de servicios de anticoncepción y planificación familiar?

    [No- pasar a la siguiente pregunta]

    [Sí- sondear]:

    a. Explíqueme cómo es una supervisión típica en las salas de planificación familiar.
    b. ¿Qué hacen los supervisores? (indagar qué preguntas suelen hacer, qué documentación suelen revisar, etc.)
    c. ¿Cómo recibe retroalimentación de la supervisión

21. ¿Algún otro organismo externo ha hecho alguna vez una supervisión sobre la prestación de servicios de anticoncepción y planificación familiar?

    [No- pasar a la siguiente pregunta]

    [Sí- sondear]:

    a. Explíqueme cómo es una supervisión típica en las salas de planificación familiar.
    b. ¿Qué hacen los supervisores? (indagar qué preguntas suelen hacer, qué documentación suelen revisar, etc.)
    c. ¿Cómo recibe retroalimentación de la supervisión

Figura S2 Guía de entrevista para personal administrativo (versión: 3 de noviembre de 2020).

Guía de entrevista para diseñadores/operadores de políticas públicas en salud reproductiva

Antecedentes:

1. Edad
2. Puesto que ocupa en este centro de salud   Tiempo en el puesto
3. Formación
4. ¿Qué tipo de capacitación en consejería anticonceptiva ha recibido? [indagar formación universitaria].

1. Cuénteme sobre su rol actual como diseñador/operador de políticas públicas en [nombre de la organización/región].

2. ¿Cómo participa usted en la prestación de servicios de anticoncepción y planificación familiar?

Gestión de la calidad

Ahora me gustaría hacerle algunas preguntas relacionadas con la consejería anticonceptiva de alta calidad y qué procesos de gestión de la calidad deben ponerse en marcha para mejorar los servicios anticonceptivos.

3. En su opinión, ¿cuál es el objetivo de la consejería y los servicios de anticoncepción y planificación familiar?

4. ¿Qué puede hacer desde su puesto para asegurarse de que las usuarias reciban servicios de anticoncepción y planificación familiar de alta calidad?

5. ¿Cuáles son las recomendaciones del gobierno respecto a la prestación de consejería e información en los servicios de salud pública?

6. ¿Qué otras partes interesadas influyen en su trabajo en políticas de planificación familiar?
   a. Por ejemplo: asociaciones civiles, ONGs, personal de salud (prestadores, administradores, etc.), academia.

7. ¿Los servicios de salud pública tienen manuales o guías de operación sobre la consejería anticonceptiva?
   a. Sondear: ¿Cuáles son los contenidos clave?

8. ¿Cómo se monitorea o evalúa el rendimiento de los prestadores de salud al proporcionar consejería anticonceptiva?

9. Cuénteme sobre algunas de las actividades de gestión de calidad que se realizan en los centros de salud de [**Ciudad de México/San Luis Potosí/Nivel federal central**].
   a. ¿Qué le gusta de este proceso?
   b. ¿Qué es lo que no le gusta?

10. ¿Qué aspectos de la consejería anticonceptiva en [**Ciudad de México/San Luis Potosí/Nivel federal central**] se beneficiarían si la calidad de los servicios mejorara? ¿Por qué?
    a. ¿Qué necesita para mejorar los servicios en anticoncepción (en la administración, la política, los puntos de entrega de servicios)?

13. ¿Alguna vez ha dado retroalimentación a prestadores de servicios de salud o Centros de Atención Médica (Centros de salud, Hospitales, Unidades de Medicina Familiar)?
    [No- pasar a la siguiente pregunta]
    [Sí]- cuénteme cómo fue este proceso [y sondear]:
    a. ¿Qué fue fácil?
    b. ¿Qué le fue difícil?

14. ¿Considera importante recibir retroalimentación de las usuarias sobre la forma en la que se proporciona la consejería anticonceptiva en el sistema público de salud?
    a. ¿Quién tendría que encargarse de recabar esta información?

15. ¿Cómo preferiría recibir esta retroalimentación (de las usuarias sobre la consejería anticonceptiva)? [De manera verbal, escrita, otra]
    a. ¿Por qué?

16. Cuénteme cómo la perspectiva de las usuarias es/podría ser incorporada a su trabajo en políticas de planificación familiar.
    a. ¿Alguna vez recibe comentarios de las usuarias sobre la satisfacción con la consejerería anticonceptiva?

17. Planteamos estas preguntas porque las encuestas de experiencia de las usuarias son una forma de conocer su percepción de los servicios de consejería anticonceptiva. ¿Cómo le parece la idea de recopilar comentarios de las usuarias a través de encuestas de experiencia para mejorar la calidad?

18. ¿Cómo podrían utilizarse los resultados de esta encuesta de las usuarias para mejorar la calidad de los servicios públicos de anticoncepción y planificación familiar?

19. Si se pudiera retroalimentar a los prestadores de servicios de salud con estos resultados, ¿preferiría entregarla por medio de comentarios individualizados (a nivel de prestador) o comentarios generales dirigidos al Centro de Salud, por Jurisdicción o por región?

20. ¿Cuál sería la mejor manera de entregar comentarios a las Unidades de Atención Médica sobre su desempeño de consejería anticonceptiva [de forma oral, escrita; en grupos, de forma

individual; recomendaciones por unidad de salud, etc.]?

   a. ¿Cómo podría este proceso ser más fácil/mejor?

21. Si las usuarias reportaran experiencias negativas respecto a la atención anticonceptiva, ¿cómo podrían abordarse esos comentarios en su centro de salud?

Figura S3 Guía de entrevistas para diseñadores/operadores de políticas públicas en salud reproductiva (versión: 3 de noviembre de 2020).

Cuadro S1 Códigos y subcódigos derivados del análisis de contenido.

**Antecedentes en planificación familiar**
Planificación familiar
Antigüedad
Capacitación
**Función actual**
General
Servicios de planificación familiar
Consejería en anticoncepción
**Discursos de autonomía**
Comunicación
Atención a las preferencias
Flexibilidad
Placer
Servicios amigables para adolescentes
Discursos de derechos
**Discursos con coerción**
Discursos coercitivos
Discursos paternalistas
**Prejuicios**
Clase social
Contexto en el que vive
Educación
Edad
Paridad
